# Genome-Wide DNA Methylation Profiling Reveals Stage- and Severity-Associated Epigenetic Alterations in Children with Posterior Urethral Valve-Associated Chronic Kidney Disease

**DOI:** 10.3390/epigenomes10030058

**Published:** 2026-09-13

**Authors:** Sachit Anand, Catherine Daise Arokiadas, Ajay Verma, Jitendra Kumar Meena, Chittaranjan Behera, Sandeep Agarwala, Kalpana Luthra

**Affiliations:** 1 Department of Pediatric Surgery, All India Institute of Medical Sciences, New Delhi 110029, India; 2 Department of Pediatrics, All India Institute of Medical Sciences, New Delhi 110029, India; 3 Department of Forensic Medicine and Toxicology, All India Institute of Medical Sciences, New Delhi 110029, India; 4 Department of Biochemistry, All India Institute of Medical Sciences, New Delhi 110029, India

**Keywords:** posterior urethral valves, obstructive uropathy, chronic kidney disease, DNA methylation, epigenomics, MethylationEPIC array, *CYP3A5*

## Abstract

Background: Chronic kidney disease (CKD) is an important cause of morbidity in children, particularly in those with congenital anomalies of the kidney and urinary tract. Although epigenetic alterations have been implicated in CKD, stage-associated genome-wide DNA methylation changes remain poorly characterized in posterior urethral valves (PUV). This study aimed to characterize DNA methylation patterns across CKD stages in children with PUV and identify potential methylation markers associated with CKD severity. Methods: Genome-wide DNA methylation profiling was performed on peripheral blood DNA from 20 boys with PUV stratified by CKD stage and 19 age-matched healthy male controls using the Illumina MethylationEPIC v2.0 array. Analyses included a global stage-wise model, stage-specific comparisons against controls and between CKD stages, an overall PUV-associated CKD-versus-control model, functional enrichment analysis, candidate CKD progression gene analysis, and CKD severity-associated methylation analysis. Results: A global stage-wise analysis identified 11,134 CpG sites showing significant methylation variation across controls and PUV-associated CKD. Differential methylation burden was limited in early CKD stages but increased markedly in advanced CKD, with 4734 and 20,242 DMPs identified in Stage 4 and Stage 5, respectively. DMP-associated genes in advanced CKD stages showed enrichment of immune-related, structural remodeling, and intracellular signaling pathways. Candidate gene analysis identified significant methylation changes in previously reported CKD progression-associated genes, with greater representation in advanced CKD stages. CKD severity-associated analysis identified cg16879596_BC21, annotated to *CYP3A5*, as positively associated with CKD severity (r = 0.74, FDR = 1.32 × 10^−5^). Methylation at this CpG site increased across CKD stages and showed strong internal discrimination between PUV-associated CKD patients and controls (AUC = 0.947, 95% CI = 0.887–1.000). Conclusions: PUV-associated CKD is characterized by stage-associated DNA methylation changes, with a marked increase in methylation burden in advanced CKD stages. The findings highlight immune, structural remodeling, and signaling pathways associated with advanced CKD and identify *CYP3A5* methylation as a potential marker of CKD severity. In future, larger longitudinal studies with independent validation and functional integration are required.

## 1. Introduction

Chronic kidney disease (CKD) is defined by abnormalities of kidney structure or function persisting for more than three months and may ultimately progress to kidney failure [1]. CKD severity is classified according to glomerular filtration rate (GFR) into Stages 1–5: Stage 1, GFR ≥ 90 mL/min/1.73 m^2^; Stage 2, 60–89 mL/min/1.73 m^2^; Stage 3a, 45–59 mL/min/1.73 m^2^; Stage 3b, 30–44 mL/min/1.73 m^2^; Stage 4, 15–29 mL/min/1.73 m^2^; and Stage 5, <15 mL/min/1.73 m^2^, according to the Kidney Disease: Improving Global Outcomes (KDIGO) criteria [2]. Although CKD is often regarded as a disease of adulthood, it remains an important cause of morbidity in children. In contrast to adult CKD, which is commonly driven by diabetes and hypertension, pediatric CKD predominantly arises from congenital anomalies of the kidney and urinary tract (CAKUT) [3,4]. Urological disorders account for a substantial proportion of CKD cases in younger children, and posterior urethral valves (PUV), a congenital obstructive uropathy affecting boys, represent an important contributor to pediatric CKD burden along with reflux nephropathy and kidney aplasia, hypoplasia, or dysplasia [5,6,7]. Long-term follow-up studies have further demonstrated the substantial burden of CKD and progression to advanced kidney disease among children born with PUV [8].

Despite advances in the diagnosis and clinical management of PUV-associated CKD, the molecular mechanisms underlying heterogeneity in kidney injury and disease progression remain incompletely understood. CKD progression is influenced by a complex interplay of genetic, environmental, developmental, and molecular factors. However, genetic variation alone does not fully explain the variability in kidney outcomes, suggesting that additional regulatory mechanisms may contribute to disease evolution. Epigenetic modifications, including DNA methylation, histone modifications, and non-coding RNAs, provide a potential link between environmental or disease-related exposures and sustained changes in gene regulation without altering the underlying DNA sequence [9]. Among these, DNA methylation is one of the most extensively studied epigenetic mechanisms.

Epigenetic modifications have been implicated in pathways relevant to CKD progression, including inflammation, fibrosis, and transforming growth factor-β signaling [10]. In a recent study, we demonstrated altered peripheral-blood global DNA methylation in children with PUV, with higher global 5-methylcytosine levels in PUV patients than the controls and exploratory associations with CKD severity and kidney scarring [11]. This provided preliminary evidence that systemic methylation alterations may be detectable in PUV-associated kidney dysfunction and supported the need for higher-resolution methylome profiling.

Despite growing evidence for epigenetic dysregulation in CKD, the stage-specific evolution of genome-wide DNA methylation changes across the CKD spectrum, particularly in pediatric cohorts with PUV, remains poorly characterized. Therefore, the present study aimed to characterize genome-wide DNA methylation patterns across CKD stages in children with PUV and to identify stage-associated methylation signatures and potential methylation markers associated with CKD severity.

## 2. Results

### 2.1. Participant Characteristics

The study cohort comprised 39 male pediatric participants, including 20 boys with PUV and 19 age-matched healthy male controls. The median age of the PUV group was 60.5 (IQR 22.8–123.0) months, while that of the control group was 60 (27.0–124.0) months. There was no significant difference in age between the two groups (*p* = 0.79, Mann–Whitney U test).

Among the PUV patients, kidney function ranged from preserved to severely impaired, based on measured GFR from DTPA radionuclide kidney scans. The median measured GFR of the PUV cohort was 64 (IQR: 23.5–85.5) mL/min/1.73 m^2^. Patients were stratified according to the KDIGO CKD stage categories. Five patients (25%) each were classified as Stage 1 and Stage 2, four patients (20%) as Stage 3, and three patients (15%) each as Stage 4 and Stage 5.

Genome-wide methylation profiling was successfully completed for all 39 participants using the Illumina Infinium MethylationEPIC v2.0 BeadChip. Post-hybridization quality assessment showed satisfactory array performance and no sample-level exclusions. Of the 936,990 CpG probes initially assessed, 865,432 probes were retained after quality control and filtering. A total of 71,558 probes (7.63%) were excluded before downstream analysis.

### 2.2. Global Stage-Associated Methylation Changes Across CKD

**Figure 1:**
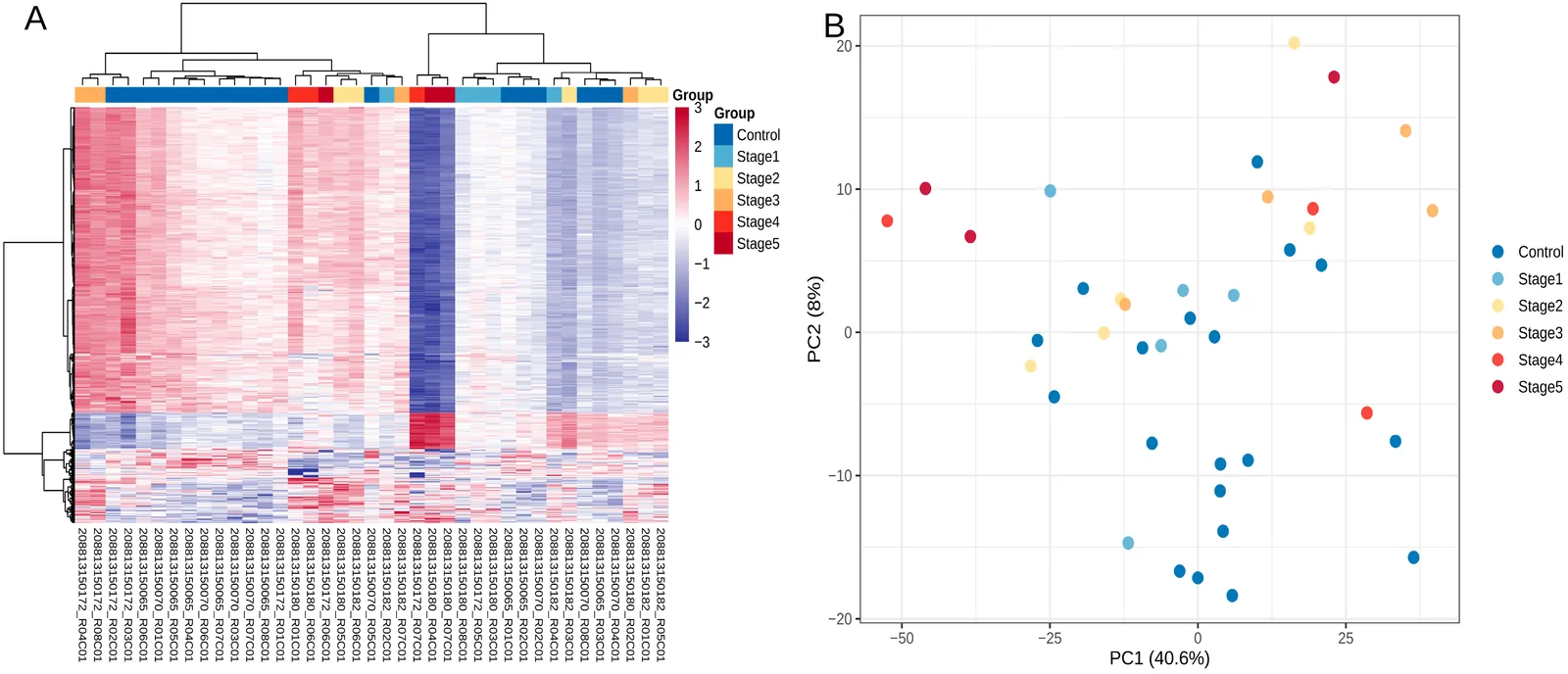
Global stage-associated methylation patterns across CKD stages. (**A**) Heatmap of the top 500 globally significant differentially methylated positions (DMPs) identified by the global moderated F-test across the controls and CKD Stages 1–5 (FDR < 0.05). Rows represent CpG sites and columns represent individual samples. Color intensity corresponds to scaled methylation levels based on Z-score-transformed β-values, with red indicating relative hypermethylation and blue indicating relative hypomethylation. Hierarchical clustering shows stage-associated methylation patterns, with greater divergence in advanced CKD stages. (**B**) Principal component analysis (PCA) of normalized β-values across all samples. Each point represents an individual sample and is colored according to control status or CKD stage. PC1 and PC2 accounted for 40.6% and 8.0% of the variance, respectively, showing partial separation of samples according to CKD stage, most apparent in advanced CKD.

The global stage-wise analysis using a moderated F-test identified 11,134 CpG sites showing significant methylation variation across the controls and CKD stages (FDR < 0.05) (Supplementary Table S1). Hierarchical clustering of the top 500 F-test significant CpGs demonstrated a stage-associated methylation pattern, with advanced CKD stages (Stages 4 and 5) showing greater divergence from the controls (Figure 1A). These findings support the presence of CKD stage-associated epigenetic variation in children with PUV.

Principal component analysis of genome-wide β-values showed partial separation according to CKD stage, again most apparent in advanced CKD (Figure 1B). QQ plot analysis showed moderate genomic inflation, with deviation from the null distribution most evident in Stage 5, consistent with a broader methylation shift in advanced disease (Supplementary Figure S1).

**Figure 2:**
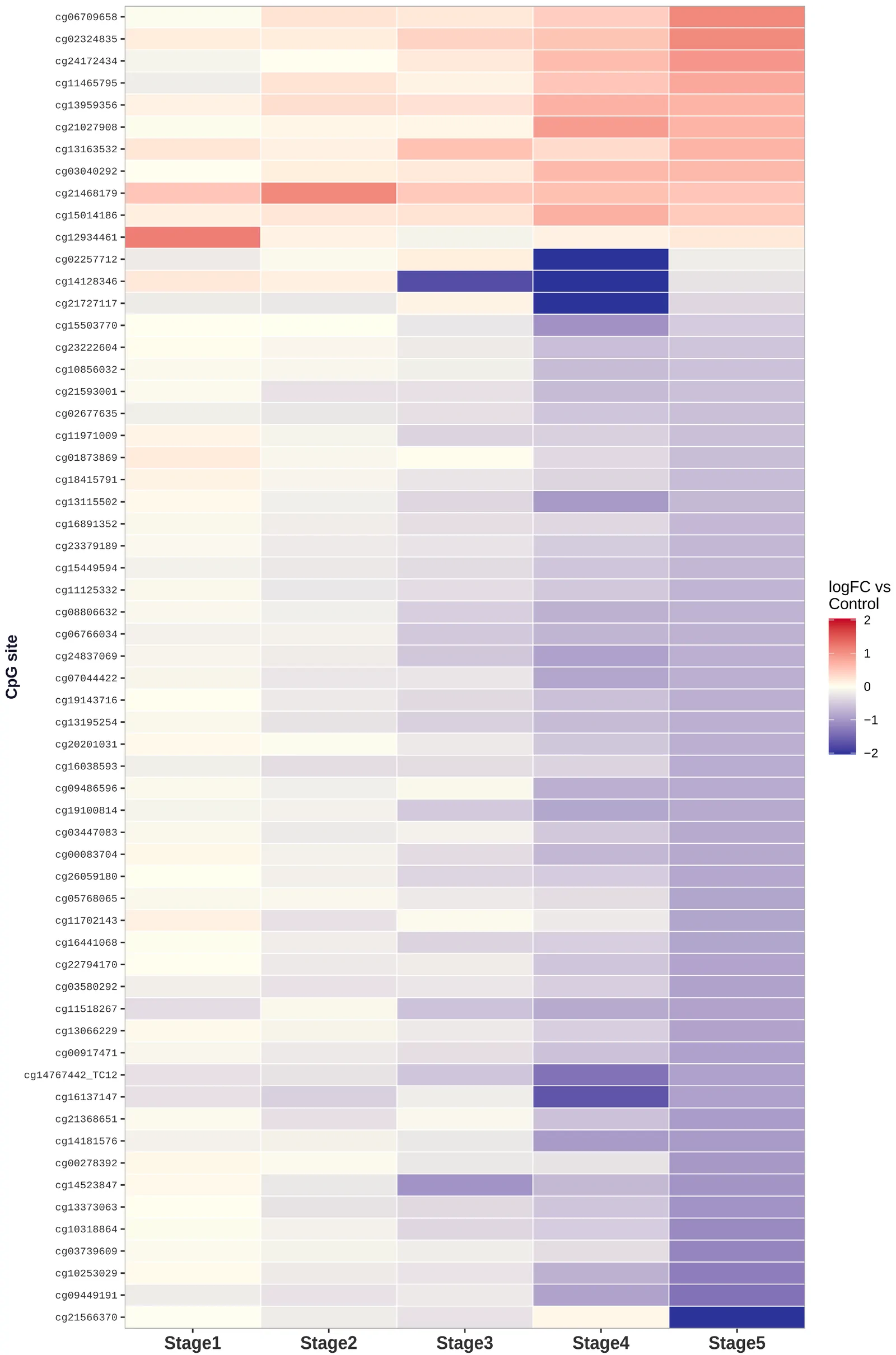
Stage-wise methylation effect pattern among globally significant CpGs. Heatmap showing stage-wise methylation effect sizes, represented as logFC values relative to the controls, for selected top CpG sites identified by the global moderated F-test across CKD stages (FDR < 0.05). Rows represent CpG sites and columns represent CKD stages compared with the controls. Red indicates relative hypermethylation and blue indicates relative hypomethylation compared with the controls. CpG sites are ordered according to the Stage 5 methylation effect size to visualize stage-associated methylation patterns, with greater deviation observed in advanced CKD stages.

To examine which stages contributed most to the global F-test signal, the top F-test significant CpGs were visualized using stage-specific logFC values relative to controls (Figure 2). Early CKD stages (Stages 1–3) showed limited methylation deviation from the controls, whereas advanced CKD stages (Stages 4–5) demonstrated pronounced bidirectional methylation shifts. This pattern suggests that the global stage-associated methylation signal was largely driven by advanced CKD stages. Therefore, stage-specific differential methylation analyses were subsequently performed to define the methylation burden and directionality within individual CKD-stage comparisons.

### 2.3. Stage-Specific Differential Methylation Analysis

**Figure 3:**
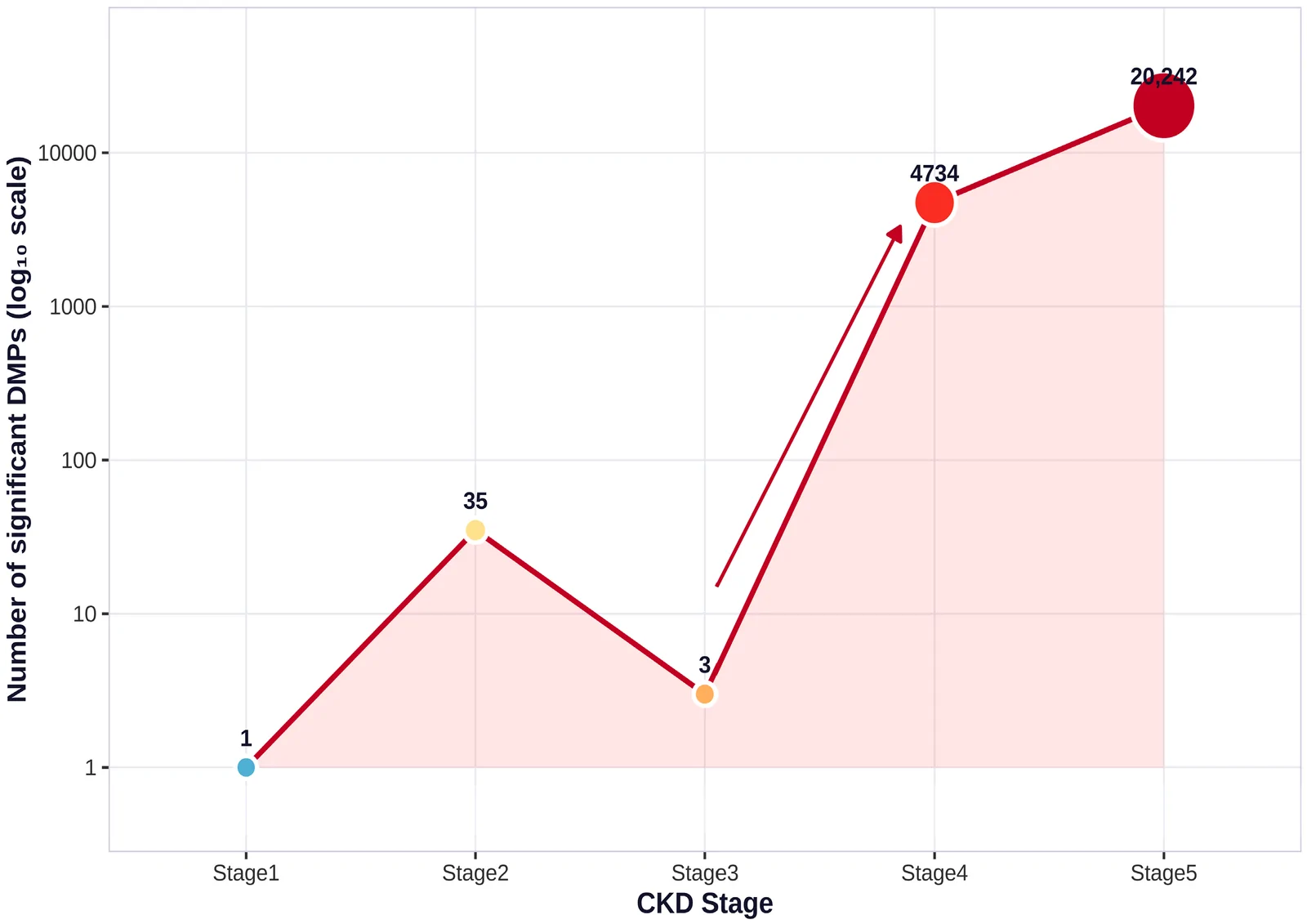
Stage-wise increase in differential methylation burden across CKD stages. Number of significant differentially methylated positions (DMPs) identified in each CKD stage compared with controls. Significant DMPs were defined using FDR < 0.05 and |logFC| > 0.2. Each point represents the number of significant DMPs for the corresponding CKD stage, displayed on a log10 scale. Point colors correspond to the respective CKD stages, with progressively darker colors representing more advanced stages. The arrow highlights the marked increase in the number of significant DMPs from Stage 3 to Stage 4. Early CKD stages showed relatively few significant DMPs, whereas Stage 4 and Stage 5 showed a marked increase in methylation burden, consistent with greater stage-associated methylation disruption in advanced CKD.

Stage-specific differential methylation analysis showed a marked increase in methylation burden with increasing CKD stage. Compared with the controls, early CKD stages showed relatively few significant DMPs: Stage 1, 1 DMP; Stage 2, 35 DMPs; and Stage 3, 3 DMPs. In contrast, advanced CKD stages showed a substantial increase in differential methylation, with 4734 DMPs in Stage 4 and 20,242 DMPs in Stage 5 (Figure 3) (Supplementary Tables S2–S6).

Analysis of methylation direction demonstrated stage-dependent changes in the balance between hypermethylated and hypomethylated CpGs. Early CKD-stage comparisons were hypermethylated, whereas advanced CKD stages showed more extensive bidirectional methylation changes (Supplementary Figure S2A). The pairwise comparison heatmap further supported this pattern, showing increasing methylation divergence across CKD stages, with the strongest separation observed in advanced CKD (Supplementary Figure S2B).

**Figure 4:**
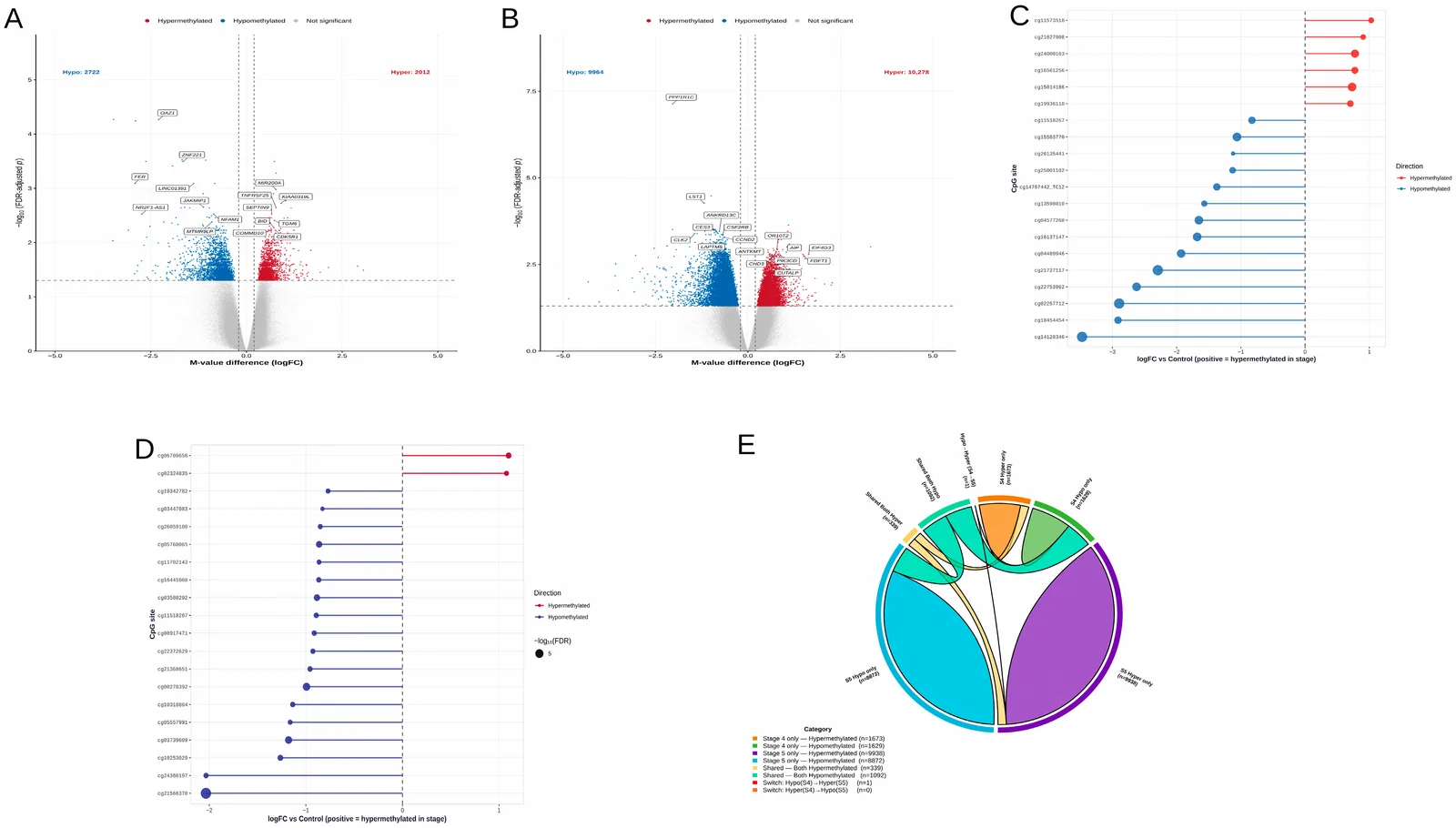
Differential methylation patterns in advanced CKD stages. (**A**) Volcano plot showing differential methylation analysis for Stage 4 CKD compared with the controls. Each point represents a CpG site. Red points indicate hypermethylated CpGs, blue points indicate hypomethylated CpGs, and gray points indicate non-significant CpGs. Selected top CpGs are annotated with corresponding gene names. (**B**) Volcano plot showing differential methylation analysis for Stage 5 CKD compared with the controls. Red points indicate hypermethylated CpGs, blue points indicate hypomethylated CpGs, and gray points indicate non-significant CpGs. Selected top CpGs are annotated with corresponding gene names. In panels A and B, the horizontal dashed line indicates the FDR-adjusted *p*-value threshold of 0.05, and the vertical dashed lines indicate the logFC thresholds of −0.2 and +0.2. (**C**) Lollipop plot showing the top significant CpGs in the Stage 4 versus control comparison, ranked by FDR. Lollipop length represents logFC based on M-value difference, and point size corresponds to −log10(FDR). Positive logFC indicates hypermethylation in Stage 4 relative to the controls, whereas negative logFC indicates hypomethylation. (**D**) Lollipop plot showing the top significant CpGs in the Stage 5 versus control comparison, ranked by FDR. Lollipop length represents logFC based on M-value difference, and point size corresponds to −log10(FDR). Positive logFC indicates hypermethylation in Stage 5 relative to the controls, whereas negative logFC indicates hypomethylation. (**E**) Chord plot showing the overlap and directionality of significant CpGs identified in Stage 4 and Stage 5 compared with the controls. Ribbon widths are proportional to the number of CpGs shared across methylation-direction categories.

Volcano plot analysis confirmed widespread methylation alterations in advanced CKD. Stage 4 showed 4734 significant DMPs, including 2722 hypomethylated and 2012 hypermethylated CpGs (Figure 4A). Stage 5 showed the highest methylation burden, with 20,242 significant DMPs, including 9964 hypomethylated and 10,278 hypermethylated CpGs (Figure 4B). These findings indicate extensive bidirectional methylation remodeling in advanced CKD, with Stage 5 showing the most prominent epigenetic alteration.

Visualization of the top 20 differentially methylated CpGs demonstrated both hypermethylated and hypomethylated loci. In Stage 4, hypomethylated CpGs showed larger effect sizes, whereas Stage 5 demonstrated a marked predominance of hypomethylated CpGs (Figure 4C,D). Manhattan plots further demonstrated that significant DMPs in Stage 4 and Stage 5 were distributed across multiple chromosomes rather than being confined to a single genomic region (Supplementary Figure S3A,B). Regional annotation showed that CpG islands were predominantly hypomethylated in both Stage 4 and Stage 5, whereas CpGs located in shelf regions and open sea regions showed relatively greater hypermethylation (Supplementary Figure S3C,D).

### 2.4. Shared Methylation Pattern Between Stage 4 and Stage 5 CKD

To identify methylation changes shared across advanced CKD, significant DMPs from Stage 4 and Stage 5 comparisons were overlapped. A substantial proportion of CpGs detected in Stage 4 were also present in Stage 5, suggesting the persistence of advanced CKD-associated methylation patterns (Figure 4E). Among the 1432 shared CpGs, 1092 were hypomethylated in both stages, while 339 were hypermethylated in both stages. The remaining one CpG showed a directional switch, changing from hypomethylation in Stage 4 to hypermethylation in Stage 5. No other CpG showed the opposite transition (Supplementary Table S7).

### 2.5. Functional Enrichment of Advanced CKD Stage-Associated DMPs

**Figure 5:**
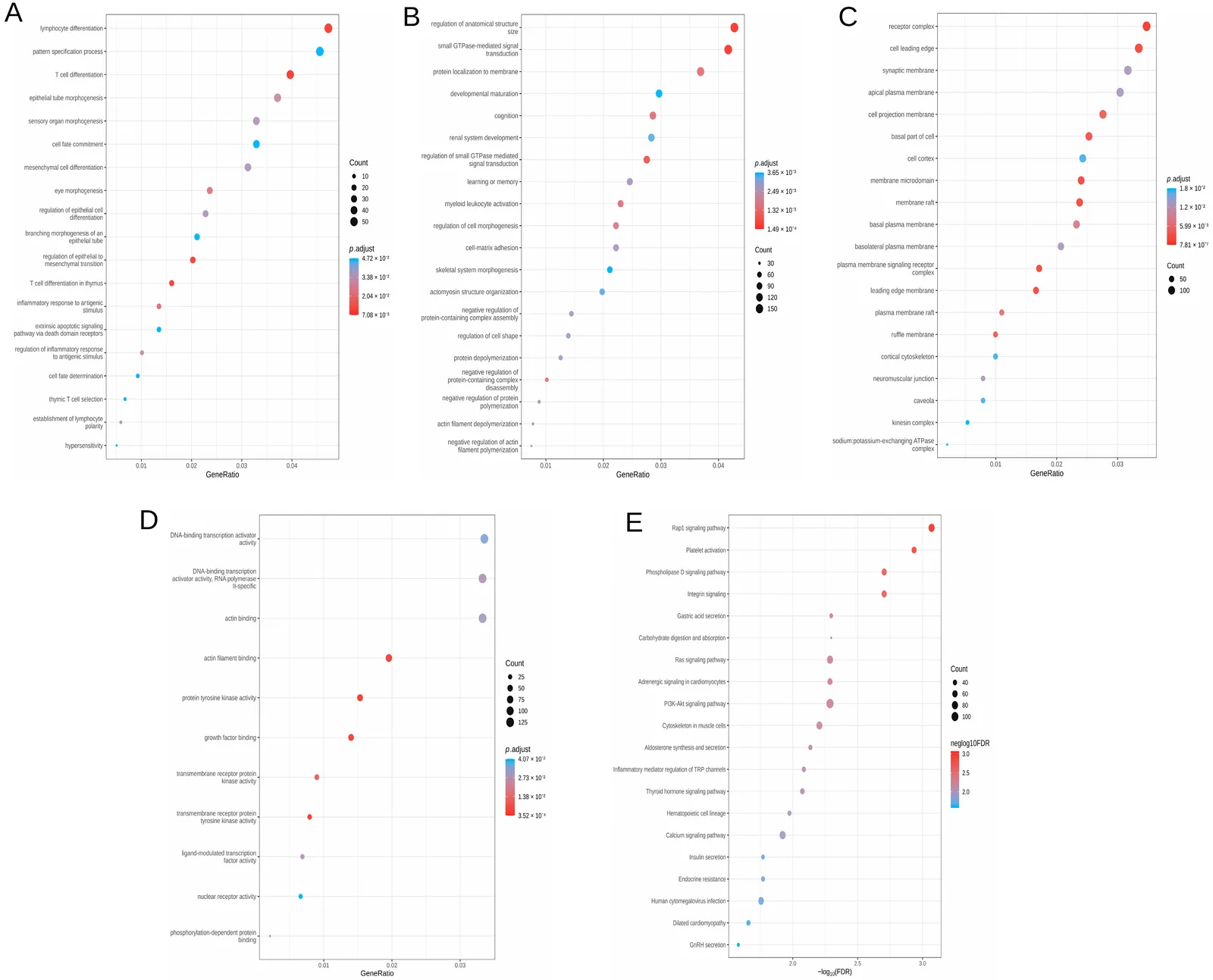
Functional enrichment of advanced CKD stage-associated DMPs. (**A**) Gene Ontology Biological Process (GO-BP) enrichment analysis of Stage 4 DMP-associated genes. Significant enrichment was limited to GO-BP terms and included immune-related processes such as lymphocyte differentiation, T-cell differentiation, inflammatory response to antigenic stimulus, and regulation of inflammatory response. (**B**) GO-BP enrichment analysis of Stage 5 DMP-associated genes. Enriched biological processes included myeloid leukocyte activation, regulation of cell morphogenesis, cell–matrix adhesion, actin filament depolymerization, and kidney system development. (**C**) Gene Ontology Cellular Component (GO-CC) enrichment analysis of Stage 5 DMP-associated genes, showing enrichment of membrane- and cytoskeleton-associated compartments, including receptor complex, cell projection membrane, and cortical cytoskeleton. (**D**) Gene Ontology Molecular Function (GO-MF) enrichment analysis of Stage 5 DMP-associated genes, showing enrichment of DNA-binding transcription factor activity, actin binding, protein tyrosine kinase activity, and nuclear receptor activity. (**E**) KEGG pathway enrichment analysis of Stage 5 DMP-associated genes, showing enrichment of the Rap1 signaling, Ras signaling, PI3K-Akt signaling, and calcium signaling pathways. Dot size represents the number of genes contributing to each enriched term, and color intensity represents the adjusted *p*-value or enrichment significance as indicated in each panel.

Functional enrichment analysis of DMGs from advanced CKD stages showed stage-dependent biological patterns. In Stage 4, significant enrichment was limited to GO Biological Process terms. Stage 4 DMP-associated genes were enriched for immune-related processes, including lymphocyte differentiation, T-cell differentiation, inflammatory response to antigenic stimulus, and the regulation of inflammatory response (Figure 5A). No significant enrichment was observed for GO Cellular Component, GO Molecular Function, KEGG, or Reactome pathway analyses in Stage 4.

In Stage 5, enrichment was broader and involved biological processes related to myeloid leukocyte activation, regulation of cell morphogenesis, cell–matrix adhesion, actin filament depolymerization, and kidney system development (Figure 5B). GO Cellular Component analysis demonstrated the enrichment of membrane- and cytoskeleton-associated compartments, including receptor complex, cell projection membrane, and cortical cytoskeleton (Figure 5C). GO Molecular Function analysis showed enrichment of DNA-binding transcription factor activity, actin binding, protein tyrosine kinase activity, and nuclear receptor activity (Figure 5D). KEGG pathway analysis identified significant enrichment of the Rap1 signaling, Ras signaling, PI3K-Akt signaling, and calcium signaling pathways (Figure 5E). These findings suggest that Stage 4 methylation changes are mainly associated with immune-related biological processes, whereas Stage 5 shows broader methylation-associated involvement of immune activation, cell adhesion, cytoskeletal organization, kidney developmental pathways, and intracellular signaling networks.

### 2.6. Overall CKD-Associated Methylation Pattern

To assess methylation differences associated with PUV-related CKD irrespective of individual stage, CKD Stages 1–5 were combined into a single group and compared with the controls. This analysis identified widespread genome-wide methylation alterations, including both hypermethylated and hypomethylated CpGs. Overall, hypermethylation predominated, with 50,926 hypermethylated CpGs and 19,298 hypomethylated CpGs (Supplementary Figure S4A) (Supplementary Table S8). Chromosomal distribution analysis showed that significant DMPs were distributed across the autosomes, without clear chromosome-specific clustering (Supplementary Figure S4B).

### 2.7. Candidate Gene-Associated Methylation Analysis

**Figure 6:**
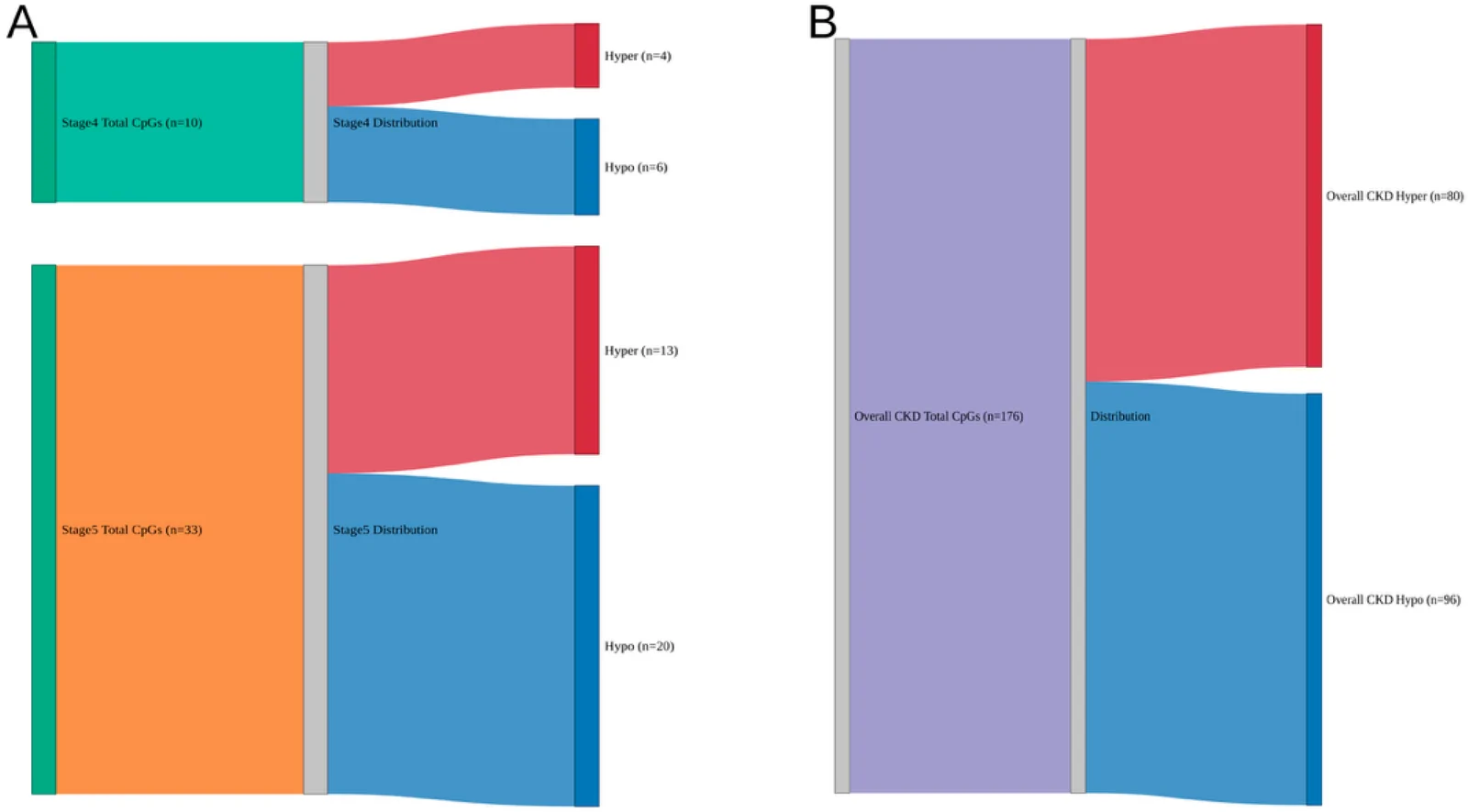
Sankey plot summarizing the distribution and methylation direction of significant DMPs mapped to candidate CKD progression-associated genes. (**A**) Across Stage 4 and Stage 5, and (**B**) across the overall CKD model. The total number of DMPs and their subdivision into subsequent hypermethylated and hypomethylated CpGs are shown. A predominance of hypomethylation was observed among both comparisons.

To evaluate whether stage-associated methylation changes involved genes previously implicated in CKD progression, a targeted candidate-gene analysis was performed using a curated panel of 110 CKD progression-associated genes (Supplementary Table S9). Significant DMPs from the stage-wise comparisons and overall CKD-versus-control were intersected with this candidate gene list. In the stage-wise analysis, no candidate gene-associated CpGs were detected in early CKD stages (Stages 1–3). Stage 4 showed 10 significant CpGs across 10 genes, including 4 hypermethylated and 6 hypomethylated CpGs, whereas Stage 5 showed 33 significant CpGs across 28 genes, including 13 hypermethylated and 20 hypomethylated CpGs (Figure 6A).

In an independent overall CKD-versus-control model, 176 significant CpGs (Supplementary Table S10) mapped to 76 candidate genes, representing 69% of the candidate-gene list. These included 80 hypermethylated CpGs and 96 hypomethylated CpGs (Figure 6B).

### 2.8. CKD Severity-Associated Methylation and Exploratory Biomarker Analysis

Correlation-based analysis of the 70,224 significant DMPs identified in the overall CKD-versus-control comparison revealed 59,027 CpGs significantly associated with ordered CKD severity (FDR < 0.05), of which 93 CpGs showed strong correlation with CKD stage (|r| ≥ 0.7) (Supplementary Table S11).

**Figure 7:**
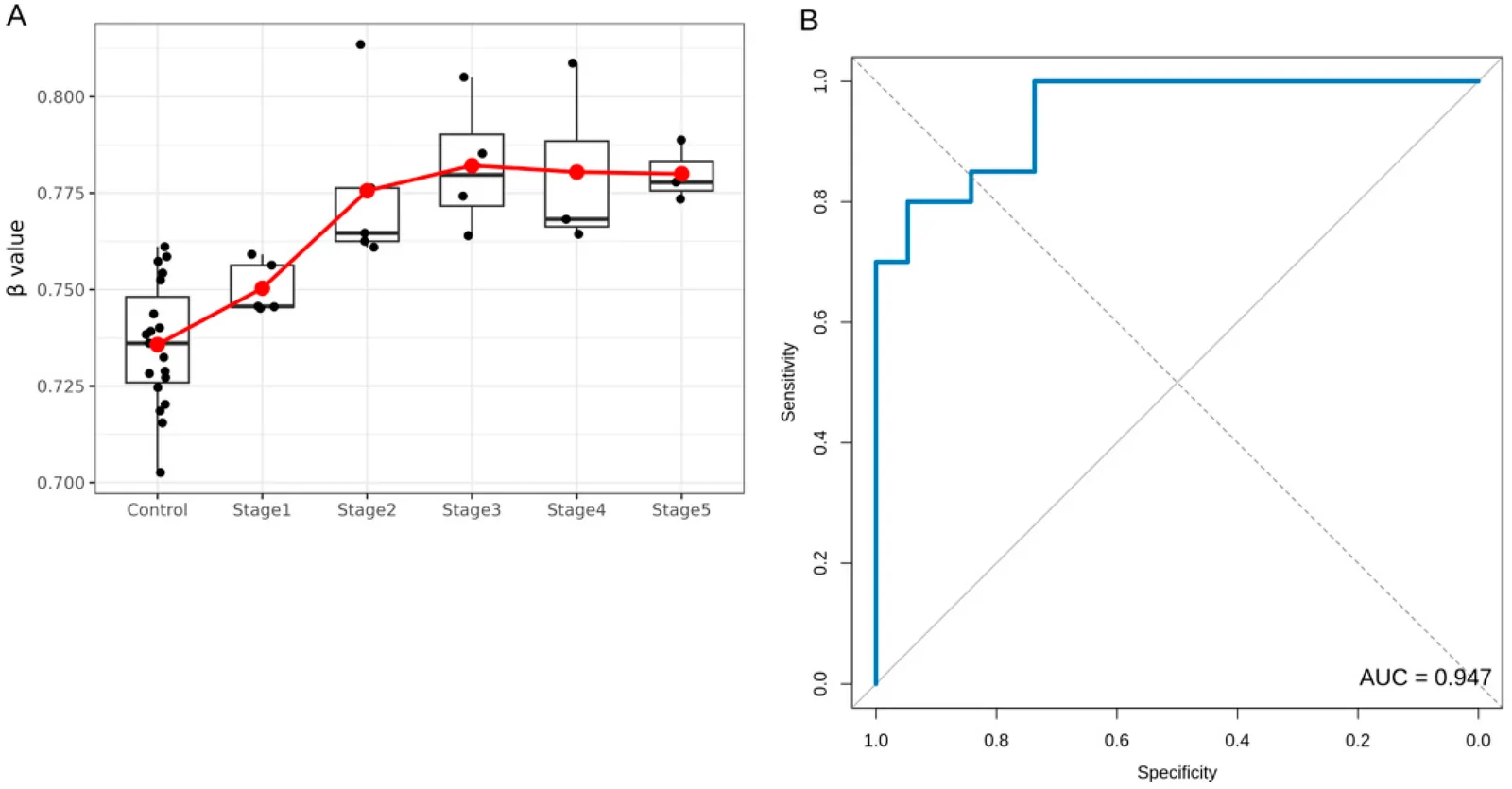
CKD severity-associated *CYP3A5* methylation and internal discriminatory performance. (**A**) Methylation β-values for cg16879596_BC21, annotated to *CYP3A5*, across the controls and CKD stages. Individual samples are shown as black dots, and boxplots represent the distribution of methylation values within each group. Red circles and connecting lines indicate group mean β-values. Methylation at cg16879596_BC21 showed an increasing pattern across CKD stages and was positively associated with CKD severity. (**B**) Receiver operating characteristic (ROC) curve for cg16879596_BC21 (*CYP3A5*) comparing PUV-associated CKD patients with the controls. The area under the curve (AUC) was 0.947 (95% CI: 0.887–1.000; DeLong method), indicating strong internal discriminatory performance.

To explore the discriminatory potential of severity-associated CpGs, ROC analysis was performed for the top-ranked CpGs from the overall CKD severity-associated analysis. cg16879596_BC21/*CYP3A5* ranked among the top three CpGs by AUC and was the highest-ranked CpG annotated to a gene, whereas the two CpGs with marginally higher AUC values were not linked to annotated genes (Table 1). Mean β-values for cg16879596_BC21 increased across groups, from 0.736 in the controls to 0.780 in Stage 5 CKD (Figure 7A). ROC analysis showed strong internal discrimination between PUV-associated CKD cases and controls, with an AUC of 0.947 (95% CI = 0.887–1.000; DeLong method) (Figure 7B). At the optimal Youden threshold (β = 0.759), cg16879596_BC21 correctly classified 16 out of 20 PUV-associated CKD patients and 18 out of 19 controls, corresponding to a sensitivity of 80.0% and specificity of 94.7%.

In the candidate-gene analysis, 149 out of the 176 candidate gene-associated CpGs were significantly associated with CKD severity (FDR < 0.05) (Supplementary Table S12). Among these, cg16879596_BC21, annotated to *CYP3A5*, showed increasing methylation with advancing CKD stage and demonstrated a strong positive correlation with CKD severity (r = 0.74; FDR = 1.32 × 10^−5^).

## 3. Discussion

This study provides a genome-wide characterization of DNA methylation changes across CKD stages in a pediatric male cohort with PUV. Using a stage-wise epigenome-wide approach, we identified 11,134 CpG sites showing significant methylation variation across the controls and PUV-associated CKD groups. Stage-specific analyses further showed that early CKD stages had limited differential methylation, whereas advanced CKD stages, particularly Stages 4 and 5, demonstrated a marked increase in methylation burden. The more than fourfold increase in significant DMPs from Stage 4 to Stage 5 suggests more extensive epigenetic remodeling in advanced disease; however, the cross-sectional design prevents direct inference of temporal accumulation. To our knowledge, this is the first study to characterize CKD stage-associated genome-wide DNA methylation patterns in children with PUV-associated CKD.

Advanced CKD is characterized by uremic toxin accumulation, oxidative stress, systemic inflammation, and immune dysregulation [12]. In the present study, Stage 4-associated methylation changes were enriched primarily in immune-related biological processes, including lymphocyte differentiation, T-cell differentiation, inflammatory response to antigenic stimulus, and the regulation of inflammatory response. This finding is consistent with previous reports showing that CKD is associated with uremic toxin-related immune dysfunction, chronic immune activation, and alterations in adaptive immune responses, including T-cell biology [13]. Because significant enrichment in Stage 4 was limited to GO Biological Process terms, these findings should be interpreted as an advanced-stage immune-associated methylation signal rather than broad pathway-level remodeling.

In contrast to Stage 4, Stage 5 showed broader methylation-associated biological enrichment, involving myeloid leukocyte activation, the regulation of cell morphogenesis, cell–matrix adhesion, actin filament depolymerization, and kidney system development. This shift from lymphocyte/T-cell-related processes in Stage 4 to myeloid activation and structural remodeling in Stage 5 may reflect increasing biological complexity in advanced kidney dysfunction. Myeloid cells are recognized contributors to kidney inflammation, fibrosis, and tissue remodeling [14], while cell–matrix adhesion and cytoskeletal pathways are central to maintaining kidney epithelial and filtration-barrier integrity [15]. Consistent with these structural and immune-related signals, KEGG analysis in Stage 5 identified enrichment of the Rap1, Ras, PI3K-Akt, and calcium signaling pathways, which are involved in cell adhesion, cytoskeletal organization, proliferation, survival, and inflammatory signaling [16,17]. Although the functional consequences of these methylation changes remain to be established, the Stage 5 enrichment pattern suggests that advanced PUV-associated CKD is accompanied by methylation changes involving immune activation, cellular architecture, and intracellular signaling networks.

Overlap analysis between Stage 4 and Stage 5 significant DMPs showed that a core subset of advanced CKD-associated methylation changes was shared across both stages, while Stage 5 showed many additional methylation alterations. Among the overlapping CpGs, 1092 were hypomethylated and 339 were hypermethylated in both Stage 4 and Stage 5, while only one CpG showed a directional switch from hypomethylation in Stage 4 to hypermethylation in Stage 5. This pattern suggests that methylation alterations observed in advanced CKD may persist across severe disease stages, whereas the larger Stage 5-specific methylation burden may reflect additional epigenetic remodeling associated with end-stage disease severity. However, due to the cross-sectional study design, these findings should be interpreted as stage-associated patterns rather than direct evidence of longitudinal methylation progression.

To evaluate methylation differences associated with PUV-related CKD irrespective of individual stage, CKD Stages 1–5 were combined and compared with the controls. This analysis showed widespread genome-wide methylation alterations, with a predominance of hypermethylated CpGs. The autosomal distribution of DMPs suggests that CKD-associated methylation changes were broadly distributed across the genome rather than confined to a specific chromosomal region. This broad genome-wide pattern contrasts with reports in which a specific epigenetic modulator preferentially alters methylation at a single chromosome; for instance, indicaxanthin, a dietary phytochemical, was shown to selectively hypomethylate chromosome 21 in a colorectal cancer cell model [18]. The absence of a comparable chromosome-specific signal in the present study may reflect the fact that peripheral blood methylation profiles integrate signals across a heterogeneous population of circulating cells, potentially averaging out chromosome-restricted effects that might be more readily detected in a homogeneous cell-line system. Whether this represents a genuine biological difference between systemic disease exposure and a discrete phytochemical stimulus, or simply the summative and subtractive effects inherent to a mixed peripheral blood cell population, remains an open question for future single-cell or cell-type-resolved methylation studies. Together with the stage-specific findings, the overall CKD analysis in the current study supports the presence of a systemic methylation signature associated with PUV-related kidney dysfunction, although the contribution of advanced CKD stages to this signal should be considered. This is consistent with previous studies suggesting that DNA methylation alterations are involved in CKD biology [19].

To assess whether the observed methylation changes involved genes previously linked to CKD progression, we performed a targeted candidate-gene analysis. Candidate gene-associated CpGs were more frequent in advanced CKD stages, particularly Stage 5, mirroring the genome-wide increase in methylation burden observed across CKD severity. The overall CKD-versus-control analysis identified significant CpGs in 76 out of 110 CKD progression-associated genes, supporting the biological relevance of the methylation signal. However, these findings should be interpreted cautiously because methylation changes were identified in peripheral blood and do not establish whether the affected genes show altered expression or directly contribute to CKD progression. Future studies integrating methylation with transcriptomic and functional data will be needed to clarify the consequences of these candidate gene-associated changes.

Among the severity-associated candidate-gene CpGs, cg16879596_BC21 annotated to *CYP3A5* emerged as the most biologically interpretable candidate marker, showing increasing methylation across CKD stages, strong positive correlation with ordered CKD severity, and high internal ROC performance. *CYP3A5* is biologically relevant because it is expressed in the kidney and has been implicated in kidney homeostatic processes, including steroid metabolism, mineralocorticoid signaling, RAAS-related regulation, sodium handling, and oxidative stress pathways [20,21]. In addition, genetic variation in *CYP3A5* has been associated with CKD progression, further supporting its relevance to kidney disease biology [22]. In the present study, cg16879596_BC21 was located in the TSS1500 region of *CYP3A5*, suggesting potential regulatory relevance. However, because gene-expression data were not available, we cannot determine whether methylation at this CpG influences *CYP3A5* transcription. Therefore, *CYP3A5* methylation at cg16879596_BC21 may be considered a potential candidate methylation marker associated with PUV-related CKD severity, requiring further validation in independent cohorts.

This study has several strengths. First, it applies genome-wide DNA methylation profiling to a clinically well-defined pediatric PUV cohort stratified by measured GFR-based CKD stages. Second, the analytical strategy combined global stage-wise testing, stage-specific pairwise comparisons, functional enrichment, advanced-stage overlap analysis, candidate CKD gene interrogation, and exploratory severity-associated marker prioritization. This layered approach allowed for the evaluation of methylation changes from both genome-wide and biologically focused perspectives. Finally, the study addresses an area in which data remain limited, namely epigenetic variation across CKD severity in children with congenital obstructive uropathy.

Several limitations should be considered when interpreting these findings. The study included a modest number of patients, particularly within individual CKD stages, which may have limited statistical power and increased sensitivity to stage-specific variability. The cross-sectional design and single-time-point methylation assessment also preclude direct inference regarding longitudinal methylation progression. DNA methylation profiling was performed in peripheral blood rather than kidney tissue, as kidney tissue sampling is not ethically justified in all cases of PUV, and is generally available only in limited clinical contexts such as nephrectomy or transplantation. Therefore, the kidney-specific relevance of these findings requires further validation. Cell-line-based models, in which epigenetic changes can be assessed under controlled, tissue-specific conditions without the confounding influence of circulating cell heterogeneity, offer a complementary strategy for validating candidate methylation markers identified in peripheral blood [23]. The cohort was limited to boys with PUV-associated CKD, and a non-PUV pediatric CKD comparison group was not included; therefore, it remains unclear whether the observed methylation patterns are specific to PUV-associated CKD or reflect broader CKD-related changes. Finally, the candidate biomarker, i.e., *CYP3A5* methylation at cg16879596_BC21 should be considered exploratory because candidate marker performance was assessed within the discovery cohort and requires validation in larger independent cohorts.

## 4. Materials and Methods

### 4.1. Study Design and Setting

This single-center, cross-sectional epigenome-wide study was performed in the Department of Pediatric Surgery, All India Institute of Medical Sciences (AIIMS), New Delhi, India, between June 2024 and May 2026. The present analysis was structured to assess DNA methylation variation across CKD stages in boys with PUV, using age-matched healthy male children as the reference group. The study protocol was approved by the Institutional Ethics Committee at our center (Ref. No: AIIMSA1432/07.06.2024, RP-5/2024; dated 18 June 2024), and all research procedures adhered to the institutional ethical guidelines. Written informed consent was obtained from the parents or legal guardians of all participants. Where appropriate, assent was also obtained from children in accordance with age-appropriate ethical standards.

### 4.2. Study Population

The study included 20 male children younger than 18 years with a confirmed diagnosis of PUV who were being followed for varying degrees of kidney dysfunction after valve ablation. PUV diagnosis was confirmed from clinical records, imaging findings, and cystoscopic documentation [24]. At recruitment, all patients were under regular follow-up in the outpatient department (OPD). These patients were stratified according to CKD stage based on measured GFR obtained from diethylenetriamine pentaacetic acid (DTPA) radionuclide kidney scan, enabling the assessment of stage-associated methylation differences [2].

Patients were excluded if they had symptomatic active urinary tract infection at the time of blood sampling, defined by compatible clinical features with a urine culture growth of >10^5^ colony-forming units/mL. Other exclusion criteria were previous kidney transplantation, additional kidney disease not attributable to PUV, or documented exposure to nephrotoxic medications during the preceding three months.

The reference group (controls) comprised 19 age-matched healthy male children recruited during the same study period. Controls were included from the general pediatric OPD at our center and had no known congenital or acquired kidney disease, no urological abnormality, no chronic systemic illness, and no previous history of UTI. At enrollment, all controls were clinically well, had no evidence of active infection, and had normal urinalysis.

### 4.3. Sample Collection and DNA Extraction

Peripheral venous blood (approximately 5 mL) was collected from each participant in sterile collection tubes. Genomic DNA was extracted from whole blood using the QIAamp DNA Blood Mini Kit (Qiagen, Hilden, Germany; Cat. No. 51106) according to the manufacturer’s protocol. DNA concentration and purity were assessed using a NanoDrop One™ spectrophotometer (Thermo Fisher Scientific, Waltham, MA, USA), and samples with A260/A280 ratios of 1.8–2.0 were taken forward for methylation profiling. Extracted DNA was aliquoted to avoid repeated freeze–thaw cycles and stored at −80 °C until bisulfite conversion and array processing.

### 4.4. Bisulfite Conversion and Methylation Array Hybridization

Genome-wide DNA methylation profiling was performed using the Infinium MethylationEPIC v2.0 BeadChip platform (Illumina, San Diego, CA, USA; Cat. No. 20087709) at an external facility. For each sample, 400 ng of genomic DNA was bisulfite-converted using the EZ DNA Methylation Kit (Zymo Research, Irvine, CA, USA; Cat. No. D5004), following the manufacturer’s instructions. The bisulfite-converted DNA was processed according to the Illumina EPIC v2.0 protocol, including amplification, enzymatic fragmentation, array hybridization, washing, and single-base extension. BeadChips were scanned using the Illumina iScan system, and raw intensity files were exported for bioinformatic analysis.

### 4.5. Bioinformatics and Statistical Analysis

#### 4.5.1. Pre-Processing and Quality Control

Raw IDAT files were analyzed in R version 4.5.3 using the minfi package version 1.56.0 [25]. Sample-level quality control was performed using detection *p*-values and signal-intensity metrics, and no samples were excluded at this stage. Background correction and normalization were performed using functional normalization implemented through the preprocessFunnorm function in minfi.

After normalization, probes were excluded if they had detection *p*-values > 0.01, mapped to sex chromosomes, overlapped with common SNPs at the CpG or single-base extension site with minor allele frequency (MAF) > 5% [26], or were known to be cross-reactive. Methylation levels were represented as β-values (ranging from 0 to 1) for visualization and biological interpretation, whereas M-values (logit-transformed β-values) were used for differential methylation testing because of their greater suitability for linear modeling.

To account for unmeasured technical or biological variation, surrogate variable analysis was performed using the sva package [27,28]. Chronological age in months was included as a covariate in full (~Group + Age) and null (~Age) models. One surrogate variable was estimated using the Leek method and incorporated, along with age, into the limma model used for downstream stage-wise analyses.

#### 4.5.2. Differential Methylation Analysis

Differential methylation analysis was performed using the empirical Bayes linear modeling framework in the limma package [29]. The design matrix included six participant categories: controls and CKD Stages 1–5, with age in months and the estimated surrogate variable included as covariates. The global stage-wise analysis used a multi-group moderated F-test to identify CpG sites showing methylation variation across the controls and CKD Stages 1–5 [30]. CpGs with an FDR < 0.05 were considered significant in this omnibus stage-wise analysis. This approach was used to detect overall stage-associated methylation heterogeneity without assigning the signal to a specific CKD stage at the initial step.

Secondary pairwise analyses were then performed to identify the stages contributing to the global methylation signal. These included comparisons of each CKD stage with the controls, as well as pairwise comparisons between CKD stages. For pairwise contrasts, CpGs were considered differentially methylated when they met both criteria: FDR < 0.05 and absolute M-value difference |logFC| > 0.2. Mean β-value differences (Δβ) were calculated as the difference between the mean β-value of each CKD-stage group and the control group to aid biological interpretation.

In an independent analysis, CKD Stages 1–5 were combined into a single PUV-associated CKD group and compared with the controls to identify methylation differences associated with CKD status irrespective of stage. Genomic inflation factors (λ) and quantile–quantile (QQ) plots were generated for all pairwise contrasts and for the overall CKD-versus-control model to assess test statistic calibration.

#### 4.5.3. Annotation

Significant differentially methylated positions (DMPs) were annotated using the IlluminaHumanMethylationEPICv2anno.20a1.hg38 annotation package, which assigns CpG probes to corresponding gene symbols and genomic features. CpG island annotations were obtained from the Illumina EPIC v2 manifest file. When a CpG probe mapped to multiple genes or transcription start site-related features, annotation was prioritized using the following hierarchy: TSS200 > TSS1500 > 5′UTR > first exon > gene body > 3′UTR > intergenic region [31]. For CpG island context, probes were prioritized as CpG island > shore > shelf > open sea [32].

For exploratory regulatory interpretation, promoter-associated DMPs were further categorized according to methylation direction. Hypomethylated CpGs located in promoter-proximal regions, including TSS200, TSS1500, 5′UTR, and first exon, were classified as potentially associated with transcriptional activation. Conversely, hypermethylated CpGs in the same promoter-associated regions were classified as potentially associated with transcriptional silencing [33]. These categories were used only for putative functional interpretation and were not considered direct evidence of altered gene expression.

#### 4.5.4. Functional Enrichment

Functional enrichment analyses were performed to interpret the biological pathways represented by stage-associated differentially methylated genes (DMGs). CpG probes were first mapped to gene symbols, and the resulting gene lists from relevant comparisons were used for enrichment testing. Gene Ontology (GO) enrichment was assessed across biological processes, molecular function, and cellular component categories using annotations from the org.Hs.eg.db database. Pathway-level enrichment was evaluated using the Kyoto Encyclopedia of Genes and Genomes (KEGG) [34] and the Reactome databases [35,36].

GO and KEGG analyses were performed using the clusterProfiler package (version 4.18.4) [37], while Reactome enrichment was carried out using ReactomePA (version 1.54.0) [38]. Enriched terms were considered significant at an FDR-adjusted *p* value < 0.05 with gene sets containing 5–2000 genes retained for downstream interpretation [39].

#### 4.5.5. Candidate Gene-Associated Methylation Analysis

To examine whether stage-associated methylation changes involved genes previously linked to CKD progression, a targeted candidate-gene analysis was performed. A curated list of 110 CKD progression-associated genes was compiled from two published studies [40,41]. Annotated significant DMPs from each stage-wise and overall CKD comparison were intersected with this candidate gene list in independent analysis.

Significant CpGs mapping to the candidate CKD progression genes were recorded for each comparison and labelled as hypermethylated or hypomethylated according to their direction of effect. These candidate gene-associated methylation changes were summarized using Sankey diagrams.

#### 4.5.6. CKD Severity-Associated Methylation and Exploratory Biomarker Analysis

To identify CpG sites showing methylation changes associated with ordered CKD severity, correlation-based analysis was performed using two CpG sets: (1) significant DMPs from the overall PUV-associated CKD vs. control comparison, and (2) CpGs mapping to the curated CKD progression candidate-gene list. CKD severity was encoded as an ordinal score: control = 0, Stage 1 = 1, Stage 2 = 2, Stage 3 = 3, Stage 4 = 4, and Stage 5 = 5.

For each CpG, standardized β-values were correlated with the CKD severity score. Pearson correlation coefficients were calculated, and *p*-values were adjusted for multiple testing using the Benjamini–Hochberg method. CpGs with an FDR < 0.05 were considered significantly associated with CKD severity, while CpGs with an absolute correlation coefficient ≥ 0.7 were classified as strongly severity-associated.

To explore the potential discriminatory value of top severity-associated CpGs, receiver operating characteristic (ROC) curve analysis was performed using the pROC package (v1.19.0.1). ROC analysis compared PUV-associated CKD cases versus controls and was used for exploratory candidate prioritization rather than clinical validation. Discriminatory performance was quantified using the area under the ROC curve (AUC), with 95% confidence intervals calculated using DeLong’s method.

## 5. Conclusions

This study demonstrates that PUV-associated CKD is accompanied by stage-associated DNA methylation changes in peripheral blood, with a marked increase in differential methylation burden in advanced CKD. While early CKD stages showed limited methylation differences, Stages 4 and 5 showed broader methylation alterations involving immune regulation, structural remodeling, and intracellular signaling pathways. Candidate-gene and severity-associated analyses further highlighted *CYP3A5* methylation at cg16879596_BC21 as a potential marker associated with CKD severity. Collectively, these findings provide initial insight into the epigenetic landscape of pediatric PUV-associated CKD and support larger longitudinal studies with independent validation and functional integration. Future work incorporating kidney cell-line models may further help establish whether the peripheral blood methylation changes identified here reflect kidney-intrinsic epigenetic alterations.

## Figures and Tables

**Table 1:** Top CKD severity-associated CpG sites ranked by internal ROC performance.

CpG	AUC	Gene	r	FDR
cg04533623_TC21	0.95789474		0.75205225	0.0002952
cg09687005_TC21	0.95789474		0.75642002	0.0002952
cg16879596_BC21	0.94736842	*CYP3A5*; *CYP3A5*; *CYP3A5*; *CYP3A5*; *CYP3A5*	0.73962758	0.00034454
cg03541909_TC21	0.93421053	*SIGLEC5*; *SIGLEC5*; *SIGLEC5*; *SIGLEC5*; *SIGLEC5*; *SIGLEC5*	0.73653455	0.00034454
cg11015143_TC21	0.93421053		0.74269229	0.00033416
cg18893854_BC21	0.93421053		0.7438787	0.00033416
cg09055750_TC21	0.92368421		0.77773037	0.00018308
cg04253779_BC21	0.92105263		0.74729884	0.0002952
cg09231403_TC21	0.91842105		0.73521724	0.00034454
cg10955164_TC21	0.91315789		0.74986144	0.0002952
cg22572517_BC21	0.91315789		0.80529809	4.4575 × 10^−5^
cg25336900_BC21	0.91052632		0.76007998	0.0002952
cg13163532_BC21	0.90526316		0.77344004	0.00018308
cg08764319_BC21	0.90263158		0.73883066	0.00034454
cg20547131_BC21	0.89473684	*MIR410*; *MIR369*; *MIR412*; *MIR409*	0.73888182	0.00034454
cg18534337_BC21	0.89210526		0.74845731	0.0002952
cg00602053_BC21	0.87631579		0.73738208	0.00034454
cg21987473_BC21	0.86578947		0.75149673	0.0002952
cg01868106_BC21	0.86315789		0.7548232	0.0002952
cg16932285_TC21	0.86315789	*C1R*; *C1R*	0.73437059	0.00034454

Abbreviations: CKD, chronic kidney disease; CpG, cytosine–phosphate–guanine; ROC, receiver operating characteristic; AUC, area under the curve; FDR, false discovery rate.

## Data Availability

The data presented in this study are included in the article and Supplementary Materials. The raw intensity files (.idat) that support the findings of this study are available upon request from the corresponding author. The R codes and scripts have been deposited in the GitHub repository (https://github.com/sachitanand-analysis/SA-Methylation-2; accessed on 7 September 2026). These are available upon request from the corresponding author.

## Supplementary Materials

The following supporting information can be downloaded at https://www.mdpi.com/article/10.3390/epigenomes10030058/s1, Supplementary Figure S1: Quantile–quantile plots for stage-specific differential methylation analyses; Supplementary Figure S2: Pairwise differential methylation burden and directionality across CKD-stage comparisons; Supplementary Figure S3: Chromosomal distribution and CpG island-context annotation of significant DMPs in advanced CKD stages; Supplementary Figure S4: Overall CKD-associated differential methylation and chromosomal distribution; Supplementary Table S1: Global stage-wise differentially methylated positions identified by moderated F-test analysis across the controls and CKD stages; Supplementary Table S2: Significant differentially methylated positions identified in Stage 1 CKD versus the controls; Supplementary Table S3: Significant differentially methylated positions identified in Stage 2 CKD versus the controls; Supplementary Table S4: Significant differentially methylated positions identified in Stage 3 CKD versus the controls; Supplementary Table S5: Significant differentially methylated positions identified in Stage 4 CKD versus the controls; Supplementary Table S6: Significant differentially methylated positions identified in Stage 5 CKD versus the controls; Supplementary Table S7: Shared differentially methylated positions identified in both Stage 4 versus the control and Stage 5 versus the control comparisons; Supplementary Table S8: Significant differentially methylated positions identified in the overall PUV-associated CKD versus the control comparison; Supplementary Table S9: CKD progression-associated candidate gene list used for targeted methylation analysis; Supplementary Table S10: Candidate gene-associated differentially methylated positions identified across overall CKD comparisons; Supplementary Table S11: CKD severity-associated CpG sites identified from significant DMPs in the overall PUV-associated CKD versus control comparison; Supplementary Table S12: CKD severity-associated CpG sites mapping to candidate CKD progression-associated genes.

## Abbreviations

The following abbreviations are used in this manuscript:

CAKUT	Congenital anomalies of the kidney and urinary tract
CI	Confidence interval
CKD	Chronic kidney disease
CpG	Cytosine–phosphate–guanine
DMG	Differentially methylated gene
DMP	Differentially methylated position
DNA	Deoxyribonucleic acid
DTPA	Diethylenetriamine pentaacetic acid
EPIC	Infinium MethylationEPIC BeadChip
FDR	False discovery rate
GFR	Glomerular filtration rate
GO	Gene Ontology
IDAT	Intensity data file
IQR	Interquartile range
KDIGO	Kidney Disease: Improving Global Outcomes
KEGG	Kyoto Encyclopedia of Genes and Genomes
MAF	Minor allele frequency
PI3K–Akt	Phosphoinositide 3-kinase–protein kinase B
PUV	Posterior urethral valves
QQ	Quantile–quantile
RAAS	Renin–angiotensin–aldosterone system
ROC	Receiver operating characteristic
SNP	Single-nucleotide polymorphism
TSS	Transcription start site
UTI	Urinary tract infection
UTR	Untranslated region
