# Supplementary material for: Genome-Wide DNA Methylation Profiling Reveals Stage- and Severity-Associated Epigenetic Alterations in Children with Posterior Urethral Valve-Associated Chronic Kidney Disease
Source: Epigenomes. 2026 Sep 13;10(3):58. doi: 10.3390/epigenomes10030058 (PMC13605981; doi:10.3390/epigenomes10030058)
Supplement: Supplementary file 1 [file epigenomes-10-00058-s001.zip › Supplementary Figures/Description of Supplementary Figures.pdf]

Supplementary Figure S1. Quantile–quantile plots for stage-specific differential methylation analyses.

(A–E) Quantile–quantile (QQ) plots showing observed versus expected p-values for differential methylation analyses of Stage 1, Stage 2, Stage 3, Stage 4, and Stage 5 CKD versus controls, respectively, after adjustment for age and surrogate variable analysis (SVA). The red dashed line represents the expected null distribution. Genomic inflation factors (λ) are indicated for each comparison. Greater deviation from the null distribution was observed in advanced CKD-stage comparisons, particularly Stage 4 and Stage 5, consistent with a higher burden of stage-associated methylation differences.

Supplementary Figure S2. Pairwise differential methylation burden and directionality across CKD-stage comparisons.

(A) Pairwise DMP count matrix showing the number of significant differentially methylated positions identified across comparisons between CKD stages and controls. Significant DMPs were defined using $FDR < 0.05$ and $|logFC| > 0.2$. Colour intensity represents the number of significant DMPs in each comparison, with darker blue indicating a higher DMP count.

(B) Directionality of significant DMPs across pairwise comparisons. Stacked bars show the proportion of hypermethylated and hypomethylated CpGs in each comparison. Red indicates hypermethylation and blue indicates hypomethylation in the numerator group of each comparison. Numbers above bars indicate the total number of significant DMPs for the corresponding comparison.

Supplementary Figure S3. Chromosomal distribution and CpG island-context annotation of significant DMPs in advanced CKD stages.

(A) Manhattan plot showing significant DMPs identified in the Stage 4 versus control comparison. Each point represents a CpG site plotted according to chromosomal position. Significant CpGs are highlighted in red above the threshold line, and selected top CpGs are labelled.

(B) Manhattan plot showing significant DMPs identified in the Stage 5 versus control comparison. Each point represents a CpG site plotted according to chromosomal position. Significant CpGs are highlighted in red above the threshold line, and selected top CpGs are labelled.

(C) Distribution of Stage 4 versus control DMPs across CpG island-related genomic features. Bars show the proportion of hypermethylated and hypomethylated DMPs within CpG islands, shores, shelves, and open sea regions. CpG islands were predominantly hypomethylated, whereas shelf and open sea regions showed relatively greater hypermethylation.

(D) Distribution of Stage 5 versus control DMPs across CpG island-related genomic features. Bars show the proportion of hypermethylated and hypomethylated DMPs within CpG islands, shores, shelves, and open sea regions. CpG islands were predominantly hypomethylated, whereas shelf and open sea regions showed relatively greater hypermethylation.

Supplementary Figure S4. Overall CKD-associated differential methylation and chromosomal distribution.

(A) Volcano plot showing differential methylation analysis for the overall PUV-associated CKD group compared with controls, after adjustment for age and surrogate variable analysis (SVA). Each point represents a CpG site. Red points indicate hypermethylated CpGs, blue points indicate hypomethylated CpGs, and grey points indicate non-significant CpGs. Selected top CpGs are annotated with corresponding gene names.

(B) Chromosomal distribution of significant DMPs identified in the overall PUV-associated CKD versus control comparison, stratified by methylation direction. Red dots indicate hypermethylated CpGs and blue dots indicate hypomethylated CpGs. Significant DMPs were distributed across autosomes without clear chromosome-specific clustering.
